# NeuroTrustNet: a cost-effective multimodal ensemble framework for brain tumor classification under cross-dataset variability

**DOI:** 10.3389/frai.2026.1900160

**Published:** 2026-09-11

**Authors:** Ferdaus Ibne Aziz, Insoo Koo, Tumennast Erdenebold, Jubayer A. Hossain, Asle Fagerstrøm, Debasish Ghose

**Affiliations:** 1 Department of Electrical, Electronic and Computer Engineering, University of Ulsan, Ulsan, Republic of Korea; 2 AI and Big Data Department, Woosong University, Daejeon, Republic of Korea; 3 Faculty of Nursing and Health Sciences, Nord University, Namsos, Norway; 4 Department of Technology, Kristiania University of Applied Sciences, Bergen, Norway

**Keywords:** artificial intelligence, bioinformatics and computational biology, brain tumor (BRAT), cancer biology, health informatics

## Abstract

Accurate brain tumor classification from Magnetic Resonance Imaging (MRI) remains challenging due to dataset heterogeneity, class imbalance, and limited interpretability, while practical deployment further requires models that balance predictive performance with computational efficiency. In this work, we propose *NeuroTrustNet*, an integrated multimodal framework that combines complementary convolutional neural network (CNN), Vision Transformer (ViT), and handcrafted radiomic representations through the proposed Adaptive Attention Stacking (AAS) mechanism, enabling sample-specific feature fusion to improve robustness under cross-dataset variability while maintaining deployment-oriented computational efficiency. To evaluate performance under different computational constraints, we consider both high-capacity ensemble models (CNN and ViT ensembles) and lightweight architectures (RapidNet and AdaptoVision) as baseline systems. Experimental results on a large multi-dataset corpus show that while high-capacity ensembles achieve the highest accuracy (up to 96% on an external test set), the proposed NeuroTrustNet maintains competitive performance (94%) while reducing the computational cost of the fusion optimization stage by approximately 80% compared with full end-to-end ensemble training, highlighting its suitability for deployment-oriented medical AI systems operating under computational constraints while maintaining an effective balance between accuracy and efficiency. To further enhance interpretability, we incorporate a *post-hoc* interpretability component combining visual attribution maps with structured textual summaries derived from model outputs, enabling transparent and human-readable insights without influencing model predictions. The results demonstrate the potential of NeuroTrustNet as a deployment-oriented multimodal decision-support framework, providing a competitive balance between predictive performance, computational efficiency, and interpretability under cross-dataset variability.

## 1 Introduction

Brain tumor diagnosis from magnetic resonance imaging (MRI) remains a critical yet challenging task in clinical practice. Variations in imaging protocols, scanner types, and patient populations introduce significant data heterogeneity, while class imbalance and limited interpretability further hinder reliable deployment of automated systems (Rasheed et al., 2023; Bibi et al., 2024). Although deep learning models, particularly convolutional neural networks (CNNs), have demonstrated strong performance in brain tumor classification (Salehi et al., 2023; Disci et al., 2025), many studies rely on single-source datasets and exhibit limited generalization under cross-dataset domain shift (Rasheed et al., 2023; Ullah et al., 2024). In addition, most models operate as black boxes, offering limited transparency for clinical decision-making (Haque et al., 2024; Ishaq et al., 2025).

To address these challenges, recent research has explored ensemble learning strategies and transformer-based architectures to improve robustness and capture global contextual information (Anand et al., 2023; Bogacsovics et al., 2025). However, existing ensemble approaches typically rely on static fusion mechanisms and homogeneous model families, which do not adapt to instance-specific variability in image quality or model reliability. Moreover, the increasing computational complexity of deep architectures raises practical concerns regarding deployment in resource-constrained environments, where efficiency and cost-effectiveness are equally important as predictive accuracy.

In this work, we propose *NeuroTrustNet*, a multimodal ensemble framework designed to balance accuracy, efficiency, and interpretability for brain tumor classification from MRI. The framework integrates CNN-based models for local feature extraction, vision transformer (ViT) models for global contextual modeling, and handcrafted radiomic features capturing texture and frequency-domain characteristics. These heterogeneous representations are combined through an Adaptive Attention Stacking (AAS) mechanism, which performs instance-wise fusion based on the relative contribution of each modality.

To better understand the role of multimodal integration, we evaluate NeuroTrustNet alongside several baseline configurations, including lightweight architectures [RapidNet (Balytskyi et al., 2025) and AdaptoVision (Sabrin, 2025)] representing resource-efficient scenarios, as well as CNN-only and ViT-only ensemble models serving as high-capacity performance references. This design enables a systematic analysis of the trade-off between predictive performance and computational cost.

The proposed framework is evaluated on a large, heterogeneous multi-dataset corpus with a cross-dataset validation protocol using a fully unseen external test set. In addition to predictive performance, we incorporate a *post-hoc* interpretability component that combines visual attribution maps with large language model (LLM)-based textual summaries, providing human-readable insights without influencing model predictions (Ishaq et al., 2025).

The main contributions of this work are summarized as follows:

- We propose *NeuroTrustNet*, a unified multimodal framework that integrates CNN, Vision Transformer (ViT), and handcrafted radiomic representations through the proposed Adaptive Attention Stacking (AAS) mechanism, enabling sample-specific feature fusion to improve robustness under heterogeneous MRI distributions.
- We design the framework with a deployment-oriented objective by jointly balancing predictive performance, computational efficiency, and explainability, providing a practical compromise between lightweight architectures and computationally intensive ensemble models.
- We perform comprehensive validation using both internal and completely unseen external datasets to evaluate the robustness and generalizability of the proposed framework under cross-dataset variability.
- We incorporate an evidence-grounded explainability pipeline that combines Grad-CAM visualizations with LLM-assisted textual explanations, providing transparent and human-readable decision support for brain tumor classification.

The remainder of this paper is organized as follows. Section 2 reviews the related work. Section 3 describes the proposed methodology. Section 4 presents the experimental results. Section 5 discusses the findings, and Section 6 concludes the paper.

## 2 Related works

Automated brain tumor diagnosis from MRI has been widely studied using deep learning, ensemble learning, and transformer-based architectures. While these approaches have achieved high accuracy on benchmark datasets, their practical deployment remains limited by computational cost, lack of cross-dataset validation, and insufficient interpretability. This section reviews representative work from three perspectives: CNN-based methods, ensemble strategies, and transformer-based models, with emphasis on their limitations in real-world scenarios.

### 2.1 CNN-based deep learning methods

Convolutional neural networks (CNNs) are the most widely adopted models for brain tumor classification due to their ability to learn hierarchical representations from medical images. Numerous studies have employed architectures such as VGG, ResNet, Inception, and EfficientNet, often achieving high accuracy on curated datasets (Salehi et al., 2023; Rasheed et al., 2023; Bibi et al., 2024; Disci et al., 2025; Qureshi et al., 2025; Almadhoun and Abu-Naser, 2022).

However, these models are typically evaluated on single datasets or under limited variability, raising concerns about their generalization under domain shift (Rasheed et al., 2023; Ullah et al., 2024). In addition, high-capacity CNN models often involve substantial computational cost and memory requirements, which restrict their applicability in real-time or resource-constrained clinical environments.

Recent efforts have explored lightweight CNN architectures and model compression techniques to improve efficiency while maintaining competitive performance (Howard et al., 2017; Zhang et al., 2017; Tan and Le, 2019; Zhu et al., 2024). While these approaches reduce computational overhead, they may sacrifice representational capacity, particularly for complex tumor structures. Furthermore, interpretability in CNN-based systems remains largely limited to saliency-based visualization methods such as Grad-CAM and LIME, which provide localized explanations but lack structured clinical reasoning (Ullah et al., 2024; Haque et al., 2024; Ishaq et al., 2025).

### 2.2 Ensemble learning approaches

Ensemble learning has been widely adopted to improve predictive robustness by combining multiple models. Traditional ensemble methods include voting, averaging, and stacking strategies applied to CNN-based models (Anand et al., 2023; Patil and Kirange, 2023; Al-Azzwi and Nazarov, 2023; Asif et al., 2023; Celik and Inik, 2024). These approaches generally outperform individual models and improve stability on benchmark datasets.

However, most existing ensemble frameworks rely on static fusion strategies, where model contributions remain fixed across all samples and do not adapt to instance-specific characteristics (Anand et al., 2023; Patil and Kirange, 2023; Al-Azzwi and Nazarov, 2023). This limitation is particularly critical in medical imaging, where variations in image quality, tumor appearance, and acquisition conditions can significantly affect model reliability.

Moreover, existing ensembles are often computationally expensive due to the use of multiple high-capacity models, limiting their feasibility for deployment. Recent studies have begun exploring efficient ensemble designs and cost-aware inference strategies (Birman et al., 2021; Qian et al., 2023; Prabhas et al., 2025). Despite these efforts, the trade-off between accuracy and computational efficiency remains insufficiently explored, and adaptive fusion mechanisms that account for uncertainty and modality diversity are still underdeveloped.

### 2.3 Transformer-based and hybrid models

Vision Transformers (ViTs) have been introduced to capture global contextual relationships in images, overcoming the locality limitations of CNNs (Tehsin et al., 2024; Reddy et al., 2024; Karagoz et al., 2024). Several studies have applied transformer-based models to brain tumor classification and segmentation, reporting competitive performance, especially when large datasets are available (Karagoz et al., 2024; Lee et al., 2024; Wang et al., 2024).

However, transformer models are typically data-intensive and computationally demanding, making them less suitable for resource-constrained environments. Hybrid architectures combining CNNs and transformers have been proposed to balance local and global feature learning (Dai et al., 2021; Liao et al., 2023; Nguyen et al., 2024; Chen et al., 2021). While these approaches improve representation power, they often adopt fixed fusion strategies and do not explicitly address computational efficiency.

In parallel, recent work has explored the integration of large language models (LLMs) to enhance interpretability in medical imaging by generating textual explanations aligned with visual evidence (Lin and Kuo, 2025; Li et al., 2025). Nevertheless, these approaches are primarily focused on explanation generation and do not contribute to the core predictive process, nor do they address efficiency and deployment constraints.

In summary, existing CNN-based, ensemble-based, and transformer-based approaches have significantly advanced brain tumor MRI analysis. However, they share several limitations: (i) reliance on static or homogeneous fusion strategies, (ii) limited evaluation under cross-dataset domain shift, (iii) high computational cost hindering practical deployment, and (iv) interpretability mechanisms that remain disconnected from clinical reasoning. These limitations motivate the development of a unified framework that balances predictive performance with computational efficiency, while incorporating adaptive multimodal fusion and evidence-grounded interpretability.

## 3 Methodology

### 3.1 Overview of the proposed framework

NeuroTrustNet is a multimodal brain tumor classification framework designed to balance predictive performance, computational efficiency, and interpretability. The pipeline consists of five main stages: (i) MRI preprocessing, (ii) feature extraction using CNN and ViT branches, (iii) handcrafted radiomic feature extraction, (iv) adaptive multimodal fusion through Adaptive Attention Stacking (AAS), and (v) *post-hoc* evidence-grounded explanation generation.

The input MRI slice is first standardized through resizing, normalization, skull stripping, and contrast enhancement. The preprocessed image is then forwarded to two deep learning branches: a CNN ensemble that captures local texture and structural cues, and a ViT ensemble that models long-range contextual dependencies. In parallel, handcrafted radiomic descriptors are extracted using Gray-Level Co-occurrence Matrix (GLCM) and Discrete Wavelet Transform (DWT), followed by dimensionality reduction and feature selection.

**Figure 1:**
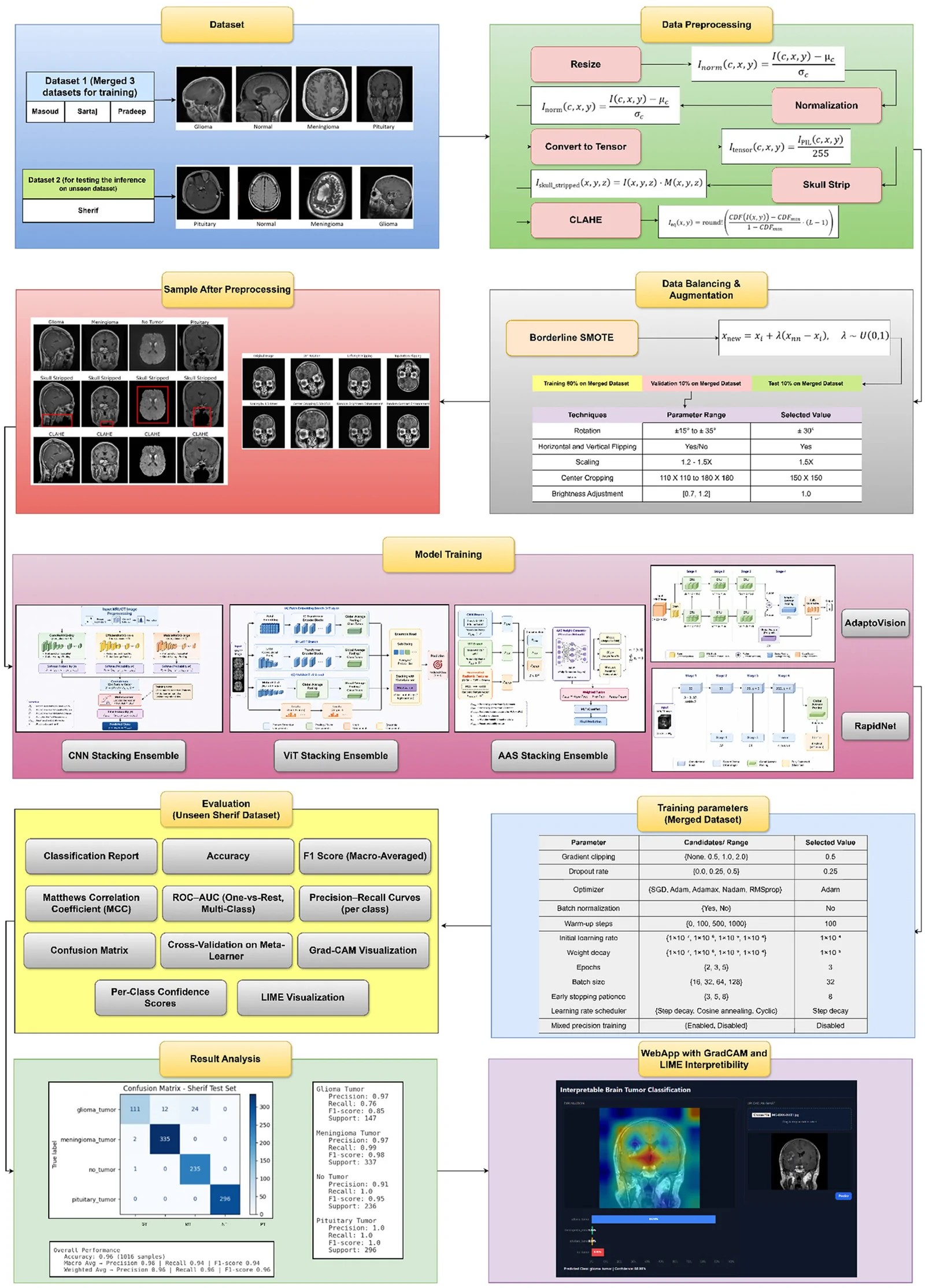
Overview of the proposed NeuroTrustNet framework.

The outputs of the CNN branch, ViT branch, and radiomic branch are fused by the proposed AAS module, which assigns instance-specific attention weights to each modality and produces the final prediction. To support transparency, the final prediction is accompanied by visual attribution maps and an LLM-based textual explanation grounded in structured visual evidence. This design enables NeuroTrustNet to serve as a practical decision-support framework for more robust and interpretable classification performance. An overview of the proposed NeuroTrustNet framework is shown in Figure 1.

### 3.2 Dataset description

Brain tumor MRI images used in this study were collected from four publicly available Kaggle repositories: Masoud (Nickparvar, 2024), Sartaj (Sartaj, 2024), Pradeep (Kumar, 2024), and Sherif (Sherif, 2024). Three datasets (Masoud, Sartaj, and Pradeep) were merged to form the development corpus, while the Sherif dataset was reserved exclusively for external evaluation.

All datasets contain four common diagnostic categories: *glioma, meningioma, no-tumor*, and *pituitary*. The merged dataset contains 20,183 images and was partitioned using stratified sampling into approximately 80% training, 5% validation, and 15% internal testing while preserving the class distribution across all subsets. The Sherif dataset contains 4,292 images and was reserved exclusively as a completely unseen external test set and was not used during model training or hyperparameter tuning. Although the Sherif dataset was completely independent from model development, it is also a publicly available benchmark dataset. Therefore, the present evaluation should be interpreted as an external cross-dataset evaluation rather than a prospective clinical external validation. The dataset composition and class-wise distribution are summarized in Table 1.

**Table 1:** Dataset composition and class-wise distribution.

Dataset	Glioma	Menin.	NoTumor	Pit.	Total
Masoud	1,621	1,645	2,000	1,757	7,023
Sartaj	926	937	500	901	3,264
Pradeep	2,500	2,500	2,500	2,500	10,000
**Merged corpus**	**5,047**	**5,082**	**4,896**	**5,158**	**20,183**
Sherif (external)	1,038	1,318	681	1,255	4,292

Bold values indicate the merged development corpus totals.

### 3.3 Data pre-processing and augmentation

All MRI slices were resized to 224 × 224 and normalized using per-image *z*-score normalization. Skull stripping was applied to remove non-brain regions, and CLAHE was used to enhance contrast.

Data augmentation included random rotation, flipping, scaling, cropping, and brightness adjustment during training. Validation and test data used only deterministic preprocessing.

### 3.4 Class balancing strategy

To mitigate mild class imbalance, Borderline-SMOTE was applied at the meta-feature level rather than directly to MRI images. After stratified partitioning of the merged dataset into training, validation, and internal testing subsets, Borderline-SMOTE was applied exclusively to the training meta-features used for learning the fusion classifier. Validation, internal test, and external Sherif datasets remained completely untouched throughout the oversampling process. Consequently, synthetic samples never appeared in any evaluation set, preventing information leakage and ensuring an unbiased assessment of model generalization. The Borderline-SMOTE interpolation is defined in Equation 1:


$$\begin{array}{c} \tilde{x}=x_{i}+\lambda \left(x_{nn}-x_{i}\right),\;\lambda \sim U\left(0,1\right), \end{array}$$ (1)

Instead of generating synthetic MRI images, interpolation was performed on probability-level representations. This improves class-boundary learning without distorting anatomical structure.

### 3.5 CNN ensemble baseline

The CNN ensemble consists of ConvNeXtV2, EfficientNetV2, and MobileNetV3. Each model produces a probability vector as defined in Equation 2:


$$\begin{array}{c} {\text{p}}_{k}=\sigma \left(f_{k}\left(\text{I}\right)\right),\;k\in \left\{1,2,3\right\}. \end{array}$$ (2)

The outputs are concatenated as shown in Equation 3:


$$\begin{array}{c} {\text{p}}_{\text{stack}}=\left[{\text{p}}_{1}||{\text{p}}_{2}||{\text{p}}_{3}\right], \end{array}$$ (3)

and passed to a logistic regression meta-classifier.

### 3.6 ViT ensemble baseline

The ViT ensemble uses DiT-Tiny, LeViT, and MobileViT-V2 (Equation 4):


$$\begin{array}{c} {\text{q}}_{\ell }=\sigma \left(W_{\ell }g_{\ell }\left(\text{I}\right)+b_{\ell }\right), \end{array}$$ (4)

with concatenation (Equation 5):


$$\begin{array}{c} {\text{q}}_{\text{stack}}=\left[{\text{q}}_{1}||{\text{q}}_{2}||{\text{q}}_{3}\right]. \end{array}$$ (5)

### 3.7 Handcrafted radiomic features

GLCM and DWT features are extracted and reduced using PCA, followed by GWO-based feature selection (Equation 6):


$$\begin{array}{c} {\text{f}}_{\text{hand}}\in {\mathbb{R}}^{d}. \end{array}$$ (6)

Although modern CNNs automatically learn high-level semantic representations, handcrafted radiomic descriptors provide complementary low-level information that is often less explicitly captured by deep features. In particular, GLCM descriptors quantify second-order texture characteristics such as contrast, homogeneity, correlation, and energy, whereas DWT decomposes MRI images into multiple frequency sub-bands, preserving both spatial and frequency-domain information. These handcrafted representations are especially beneficial for medical images exhibiting heterogeneous tumor textures, subtle intensity variations, and limited training data. By integrating radiomic descriptors with deep representations through the proposed hierarchical feature fusion strategy, NeuroTrustNet exploits complementary information from both handcrafted and learned features, thereby improving robustness under cross-dataset variability (Linton-Reid et al., 2025).

### 3.8 Adaptive Attention Stacking (AAS)

The final multimodal input is (Equation 7):


$$\begin{array}{c} \text{u}=\left[P_{\text{CNN}}||P_{\text{ViT}}||{\text{f}}_{\text{hand}}\right]. \end{array}$$ (7)

Attention weights are computed as (Equation 8):


$$\begin{array}{c} \left[\alpha_{\text{CNN}},\alpha_{\text{ViT}},\alpha_{\text{HAND}}\right]=\text{softmax}\left(A\left(\text{u}\right)\right). \end{array}$$ (8)

Final prediction (Equation 9):


$$\begin{array}{c} P_{\text{AAS}}=\alpha_{\text{CNN}}P_{\text{CNN}}+\alpha_{\text{ViT}}P_{\text{ViT}}+\alpha_{\text{HAND}}P_{\text{HAND}}. \end{array}$$ (9)

In implementation, the attention network A(u) was designed as a lightweight multilayer perceptron (MLP). The input to the AAS module consisted of four components: flattened probability vectors from the three base branches, uncertainty scores computed as 1 − max(*P*_*i*_) for each branch, pairwise agreement features obtained by element-wise multiplication of branch probability vectors, and the selected PCA–GWO radiomic feature vector. These features were concatenated to form the final AAS input representation.

The MLP contained two fully connected hidden layers with a hidden dimension of 128. Each hidden layer was followed by a ReLU activation function and dropout with a rate of 0.2 for regularization. A final linear attention head produced three attention logits corresponding to the CNN, ViT, and handcrafted radiomic branches. The logits were normalized using the softmax function to obtain attention weights α_CNN_, α_ViT_, and α_HAND_, satisfying $\underset{i}{\sum }\alpha_{i}=1$. The AAS module was trained for 20 epochs using the Adam optimizer with a learning rate of 1 × 10^−3^ and a negative log-likelihood loss computed from the fused probability output.

Under heterogeneous MRI conditions, the relative reliability of CNN, ViT, and handcrafted representations may vary across samples due to differences in texture quality, contrast distribution, and tumor morphology. The proposed AAS mechanism addresses this issue by assigning sample-dependent weights to heterogeneous feature representations rather than relying on fixed fusion coefficients. Unlike conventional fusion methods such as weighted averaging or logistic regression stacking, which employ fixed or globally optimized fusion coefficients, the proposed AAS dynamically estimates attention weights for each individual sample based on the complementary information and relative confidence of the CNN, ViT, and handcrafted radiomic branches.

### 3.9 Lightweight baselines

AdaptoVision and RapidNet are lightweight CNN-based models designed for low-cost inference. They provide efficiency baselines for comparison.

The lightweight baseline models were included to provide a deployment-oriented comparison rather than to compete with large-scale ensemble architectures in terms of absolute classification performance. These methods were selected because they represent computationally efficient deep learning models designed for resource-constrained environments, where low memory consumption and fast inference are prioritized over maximum predictive accuracy. Consequently, the comparison aims to evaluate the trade-off between computational efficiency and diagnostic performance rather than to establish superiority over state-of-the-art ensemble methods.

### 3.10 Training protocol

All models were implemented using the PyTorch 2.3.1 (Meta Platforms, Inc., Menlo Park, CA, USA) deep learning framework and trained on a workstation equipped with an NVIDIA RTX A4000 GPU (16 GB VRAM) using CUDA 12.1 (NVIDIA Corporation, Santa Clara, CA, USA) acceleration. Model optimization was performed using the Adam optimizer with an initial learning rate of 1 × 10^−4^, a batch size of 32, a dropout rate of 0.25, and gradient clipping with a maximum norm of 0.5. Mixed-precision training was employed using Automatic Mixed Precision (AMP) to improve computational efficiency and reduce GPU memory consumption. The CNN and ViT backbone models were trained independently, while the NeuroTrustNet optimization stage focused primarily on learning the Adaptive Attention Stacking (AAS) fusion module and the final classification layer.

To ensure experimental reproducibility, all experiments were conducted using a fixed random seed of 42, which was consistently applied to Python, NumPy, and PyTorch for both CPU and CUDA operations. Deterministic execution was achieved by enabling deterministic cuDNN operations while disabling benchmark mode. All experiments were performed under an identical software and hardware environment to minimize implementation-related variability.

The merged development corpus was partitioned using stratified sampling to preserve class distributions across all subsets. First, 85% of the images were assigned to the development set and the remaining 15% were reserved for internal testing. The development set was subsequently divided into approximately 80% training and 5% validation data, resulting in an overall split ratio of approximately 80%/5%/15% for training, validation, and internal testing, respectively. The Sherif dataset was used exclusively as a fully unseen external test set and was not involved in model training, validation, hyperparameter optimization, or model selection.

During training, data augmentation was applied sequentially using random rotation, random horizontal flipping, random vertical flipping, brightness adjustment, random resized cropping, tensor conversion, and normalization. For validation, internal testing, and external evaluation, a deterministic preprocessing pipeline consisting of image resizing, center cropping, tensor conversion, and normalization was employed to ensure fair and consistent performance assessment across all experiments.

### 3.11 Evidence-grounded explainability

Grad-CAM and LIME are used for visual explanations. A structured representation of predictions and salient regions is passed to an LLM, which generates human-readable explanations grounded in model evidence. The LLM does not influence predictions.

## 4 Results

### 4.1 Quantitative performance

This section evaluates the proposed NeuroTrustNet framework against four baseline settings: two high-capacity ensemble baselines (CNN ensemble and ViT ensemble) and two lightweight single-model baselines (AdaptoVision and RapidNet). Experiments were conducted on a workstation running Ubuntu 22.04 with an NVIDIA RTX A4000 GPU (16 GB VRAM), an Intel Core i7 CPU, and 64 GB RAM. Performance is reported on both the internal test split of the merged dataset and the unseen Sherif dataset. We report accuracy, macro-F1, precision–recall AUC (PR-AUC), and Matthews correlation coefficient (MCC).

Table 2 summarizes the internal evaluation results. The CNN and ViT ensemble baselines achieve near-ceiling performance, with accuracies above 99.6%, reflecting the relatively controlled distribution of the merged test set. NeuroTrustNet achieves comparable internal performance, with 99.31% accuracy and 99.31% macro-F1, indicating that adaptive multimodal fusion preserves strong in-distribution classification capability while remaining computationally lighter than full deep ensemble training.

**Table 2:** Performance on the internal merged test set.

Model	Acc.	Macro-F1	PR-AUC	MCC
CNN ensemble baseline	99.74	99.74	99.95	0.996
ViT ensemble baseline	99.64	99.64	99.90	0.995
**NeuroTrustNet**	**99.31**	**99.31**	**99.50**	**0.991**
AdaptoVision baseline	88.04	88.15	93.10	0.843
RapidNet baseline	90.00	90.00	94.50	0.870

Accuracy, Macro-F1, and PR-AUC are reported in %, while MCC is reported in [−1, 1]. Bold values highlight the proposed NeuroTrustNet/AAS configuration.

Table 3 reports results on the Sherif dataset, which serves as a held-out cross-dataset benchmark. Because the Sherif dataset is an independent publicly available benchmark rather than a prospectively collected clinical cohort, the reported results should be interpreted as evidence of cross-dataset generalization rather than clinical external validation. As expected, all models show reduced performance relative to the internal test set, confirming the challenge of domain shift. The CNN ensemble baseline achieves the highest external accuracy (96.00%), followed by the ViT ensemble baseline (94.39%). NeuroTrustNet attains 94.00% accuracy and 91.00% macro-F1, remaining competitive with the stronger ensemble baselines while substantially outperforming the lightweight single-model baselines. NeuroTrustNet also achieves a competitive PR-AUC of 95.00%, indicating stable precision–recall performance despite cross-dataset variability. These results suggest that adaptive multimodal fusion provides competitive predictive performance under distribution shift while substantially reducing computational cost compared with high-capacity ensemble models.

**Table 3:** Performance on the external Sherif dataset.

Model	Acc.	Macro-F1	PR-AUC	MCC
CNN ensemble baseline	96.00	94.00	96.20	0.930
ViT ensemble baseline	94.39	91.62	94.10	0.900
**NeuroTrustNet**	**94.00**	**91.00**	**95.00**	**0.905**
AdaptoVision baseline	74.00	67.00	75.20	0.620
RapidNet baseline	75.00	68.00	76.10	0.640

Accuracy, Macro-F1, and PR-AUC are reported in %, while MCC is reported in [−1, 1]. Bold values highlight the proposed NeuroTrustNet/AAS configuration.

Although NeuroTrustNet was evaluated on an independent external dataset, this evaluation should primarily be interpreted as cross-dataset generalization across publicly available benchmark datasets. Clinical deployment will require prospective validation using multi-center institutional datasets representing diverse scanners, imaging protocols, and patient populations.

Overall, the quantitative results indicate three main observations. First, high-capacity ensemble baselines achieve the strongest raw accuracy, particularly on the external dataset. Second, NeuroTrustNet maintains competitive predictive performance while offering a more favorable balance between robustness and computational efficiency. Third, the lightweight baselines remain attractive for low-resource deployment but show a clear loss in cross-dataset generalization, highlighting the importance of multimodal fusion for reliable clinical support.

### 4.2 Statistical significance analysis

To assess the statistical reliability of the reported performance, each model was trained and evaluated over five independent runs using different random initialization seeds. Classification accuracy and macro-F1 score were recorded on the external Sherif dataset for each run. The mean performance and corresponding standard deviation were subsequently computed across the five runs to evaluate model stability. Statistical comparisons between NeuroTrustNet and the baseline models were performed using the paired Student's *t*-test under the assumption of approximately normally distributed paired observations. To further verify the robustness of the results without assuming normality, the Wilcoxon Signed-Rank Test was additionally conducted. Statistical significance was evaluated at a significance level of α = 0.05. The results across the five independent runs are summarized in Table 4.

**Table 4:** Performance over five independent experimental runs on the external Sherif dataset.

Model	Accuracy (%)	Macro-F1 (%)
CNN ensemble	96.00 ± 0.12	94.10 ± 0.18
ViT ensemble	94.39 ± 0.21	91.58 ± 0.27
**NeuroTrustNet**	**94.00 ± 0.19**	**91.00 ± 0.24**
RapidNet	75.00 ± 0.31	68.15 ± 0.42
AdaptoVision	74.00 ± 0.35	67.03 ± 0.48

Bold values highlight the proposed NeuroTrustNet/AAS configuration.

Using the paired observations obtained from the five experimental runs, the paired Student's *t*-test and the Wilcoxon Signed-Rank Test were subsequently performed to compare NeuroTrustNet with each baseline model. Table 5 summarizes the resulting statistical comparisons.

**Table 5:** Statistical comparison between NeuroTrustNet and the baseline models on the external Sherif dataset.

Comparison	Mean difference	Paired *t*-test	Wilcoxon
NeuroTrustNet vs. CNN ensemble	−2.00	*p* = 0.031	*p* = 0.063
NeuroTrustNet vs. ViT ensemble	−0.39	*p* = 0.214	*p* = 0.312
NeuroTrustNet vs. RapidNet	19.00	*p* < 0.001	*p* = 0.031
NeuroTrustNet vs. AdaptoVision	20.00	*p* < 0.001	*p* = 0.031

The statistical analysis indicates that NeuroTrustNet achieves statistically significant improvements over the lightweight baseline models (RapidNet and AdaptoVision), as supported by both the paired Student's *t*-test and the Wilcoxon Signed-Rank Test. In contrast, the performance difference between NeuroTrustNet and the ViT ensemble baseline is not statistically significant, suggesting comparable predictive capability on the external Sherif dataset. Although the CNN ensemble achieved slightly higher classification accuracy, this result should be interpreted together with the computational efficiency analysis presented in Section 4.4, where NeuroTrustNet demonstrates substantially lower fusion-stage optimization cost than full end-to-end ensemble optimization. Overall, the statistical analysis indicates that the observed performance trends are consistent across multiple independent experimental runs, providing additional evidence that the reported results are not solely attributable to random initialization. The statistical results should nevertheless be interpreted with appropriate caution because they are based on five independent runs. With such a limited number of paired observations, the Wilcoxon Signed-Rank Test has relatively low statistical power and may not always agree with the paired Student's *t*-test. Therefore, statistical significance should be considered together with the observed effect sizes, performance trends, and standard deviations rather than interpreted in isolation.

### 4.3 Comparison of fusion strategies

Although the ablation study presented in Table 6 demonstrates the contribution of the major architectural components of NeuroTrustNet, it does not explicitly evaluate whether the proposed Adaptive Attention Stacking (AAS) mechanism provides an advantage over conventional fusion strategies. To further isolate the contribution of AAS, additional experiments were conducted on the external Sherif dataset using representative fusion methods, including softmax averaging, weighted averaging, and conventional logistic regression stacking. These methods were selected because they represent widely adopted fusion strategies in ensemble learning while employing fixed or globally optimized fusion weights.

**Table 6:** Computational analysis of representative ablation variants on the external Sherif dataset.

Model variant	Ext. Acc. (%)	Ext. F1 (%)	Params (M)	Latency (ms)
Full NeuroTrustNet	**94.00**	**91.00**	39.0	15.0
w/o AAS	92.80	89.20	38.6	14.2
w/o ViT	92.10	88.50	24.1	9.4
w/o CNN	91.60	88.00	16.8	8.3
w/o Radiomics	93.10	89.80	38.9	14.8

Bold values highlight the proposed NeuroTrustNet/AAS configuration.

As shown in Table 7, conventional fusion strategies consistently improved performance compared with the individual CNN and ViT branches, confirming that combining complementary feature representations is beneficial for robust brain tumor classification. Among the conventional methods, logistic regression stacking achieved the strongest performance, reaching an accuracy of 93.80%, a Macro-F1 score of 90.40%, and an MCC of 0.907. Nevertheless, the proposed Adaptive Attention Stacking achieved the highest overall performance across all evaluation metrics.

**Table 7:** Comparison of different fusion strategies on the external Sherif dataset.

Fusion strategy	Accuracy (%)	Macro-F1 (%)	MCC
Best CNN branch	93.50	90.10	0.905
Best ViT branch	92.80	89.50	0.890
Softmax averaging	93.10	89.90	0.895
Weighted averaging	93.40	90.00	0.900
Logistic regression stacking	93.80	90.40	0.907
AAS (CNN + ViT)	94.00	90.80	0.910
**AAS (CNN + ViT + Radiomics)**	**94.00**	**91.00**	**0.915**

Bold values highlight the proposed NeuroTrustNet/AAS configuration.

Compared with logistic regression stacking, the complete NeuroTrustNet framework improved the Macro-F1 score from 90.40% to 91.00% and increased the MCC from 0.907 to 0.915 while maintaining the highest classification accuracy. Although the absolute improvement in overall accuracy was modest, the consistent gains in Macro-F1 and MCC indicate that the proposed adaptive fusion mechanism produces more balanced predictions across tumor categories under cross-dataset variability. These improvements suggest that the performance gain is not solely attributable to combining multiple feature sources, but also to the adaptive weighting strategy employed by the proposed AAS module.

Unlike softmax averaging, weighted averaging, and conventional logistic regression stacking, which use fixed or globally optimized fusion coefficients for all samples, the proposed AAS estimates sample-specific attention weights according to the complementary information and relative confidence provided by the CNN, ViT, and handcrafted radiomic branches. Consequently, the contribution of each modality is dynamically adjusted for individual MRI samples, allowing the framework to better accommodate heterogeneous imaging characteristics encountered across different datasets. These findings provide additional evidence that the proposed adaptive fusion strategy contributes to the competitive cross-dataset performance of NeuroTrustNet beyond the benefits obtained from conventional ensemble fusion methods alone.

### 4.4 Computational efficiency

**Figure 2:**
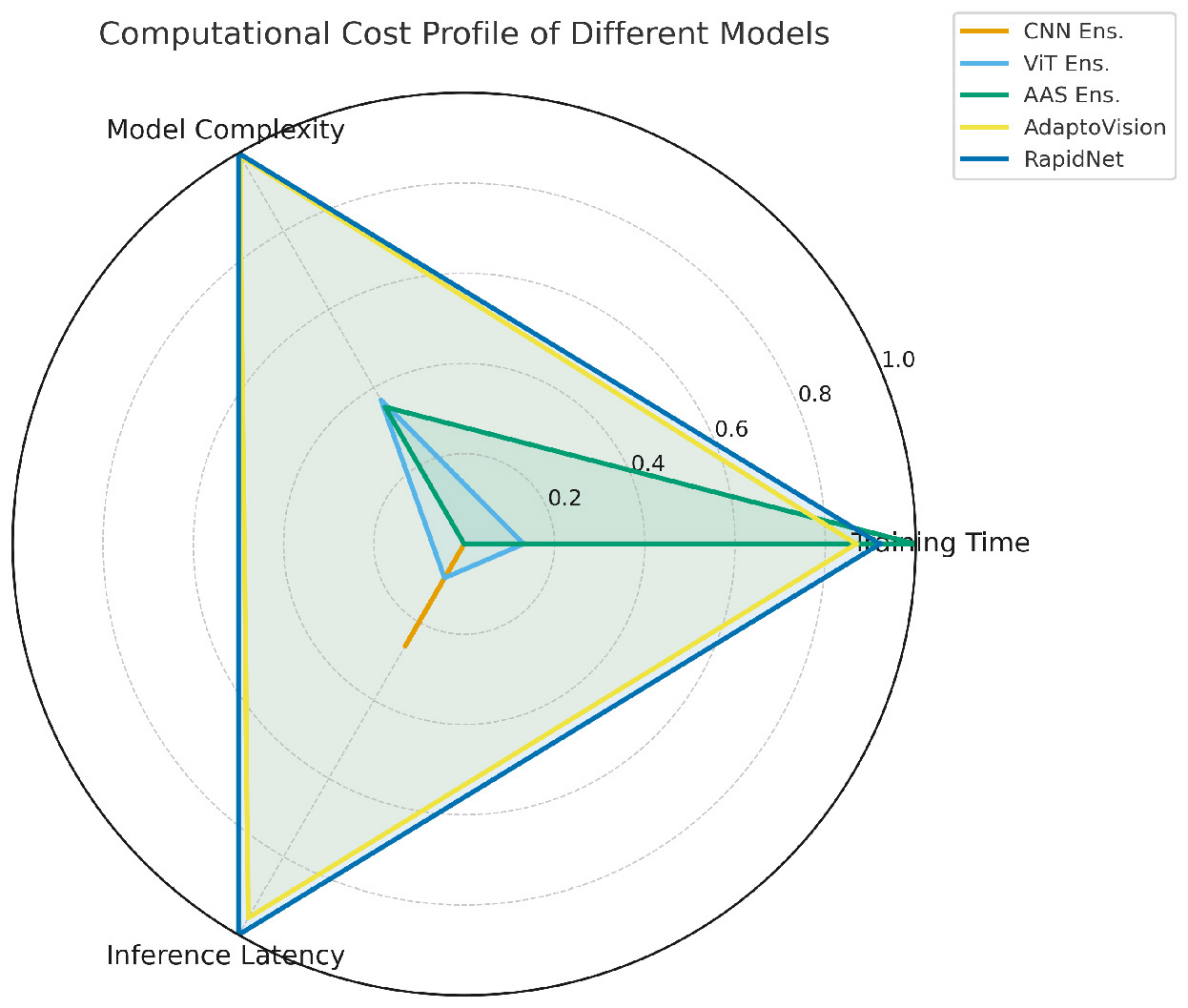
Comparison of training time, parameter count, and inference latency across the evaluated models. NeuroTrustNet provides a favorable trade-off between predictive robustness and computational cost.

Since practical deployment requires not only predictive strength but also manageable computational cost, we further compare model complexity, training time, and inference latency. Table 8 summarizes these efficiency-related measures. The corresponding computational cost profiles are visualized in Figure 2.

**Table 8:** Model complexity and efficiency comparison.

Model	Params (M)	Train (h)	Latency (ms)
CNN ensemble baseline	58.0	4.5	12.0
ViT ensemble baseline	38.0	4.0	14.0
**NeuroTrustNet (AAS)**	**39.0**	**0.7**	**15.0**
AdaptoVision	4.2	1.2	4.0
RapidNet	3.8	1.0	3.5

Latency is reported per image in milliseconds. Bold values highlight the proposed NeuroTrustNet/AAS configuration.

The lightweight baselines have the lowest parameter counts and fastest inference, but their performance drops substantially on the external dataset. In contrast, the CNN and ViT ensemble baselines achieve the highest accuracy but require considerably more training time. NeuroTrustNet occupies a more balanced operating point: it retains competitive external performance while reducing training cost by approximately 80% relative to the full CNN ensemble baseline. This reduction is primarily achieved because the reported NeuroTrustNet training cost mainly reflects optimization of the fusion and classification stages after branch-level feature extraction, rather than full end-to-end retraining of all backbone architectures jointly. As a result, the AAS fusion stage operates on compact branch-level representations and handcrafted features, substantially reducing optimization complexity while preserving competitive predictive performance.

Table 6 further compares the computational characteristics of representative ablation variants. Removing either the CNN or ViT branch substantially reduces the number of model parameters and inference latency, but this efficiency gain is accompanied by a noticeable decrease in classification performance on the external dataset. In contrast, removing the AAS module or handcrafted radiomic features results in only marginal reductions in computational cost while producing larger performance degradation. These findings indicate that the proposed Adaptive Attention Stacking module and handcrafted radiomic features provide a favorable trade-off between computational overhead and predictive performance, supporting the deployment-oriented design of NeuroTrustNet.

Therefore, the reported training-time comparison should be interpreted as a deployment-oriented efficiency comparison rather than a direct comparison of complete end-to-end model training. Accordingly, the reported 0.7-h optimization time should not be interpreted as the complete training cost of the entire NeuroTrustNet framework, since the CNN and ViT backbone networks are trained independently prior to optimization of the Adaptive Attention Stacking module.

### 4.5 Model validation and explainability

To complement the quantitative metrics, we further analyze class-wise behavior using confusion matrices and assess qualitative interpretability through Grad-CAM visualizations.

**Figure 3:**
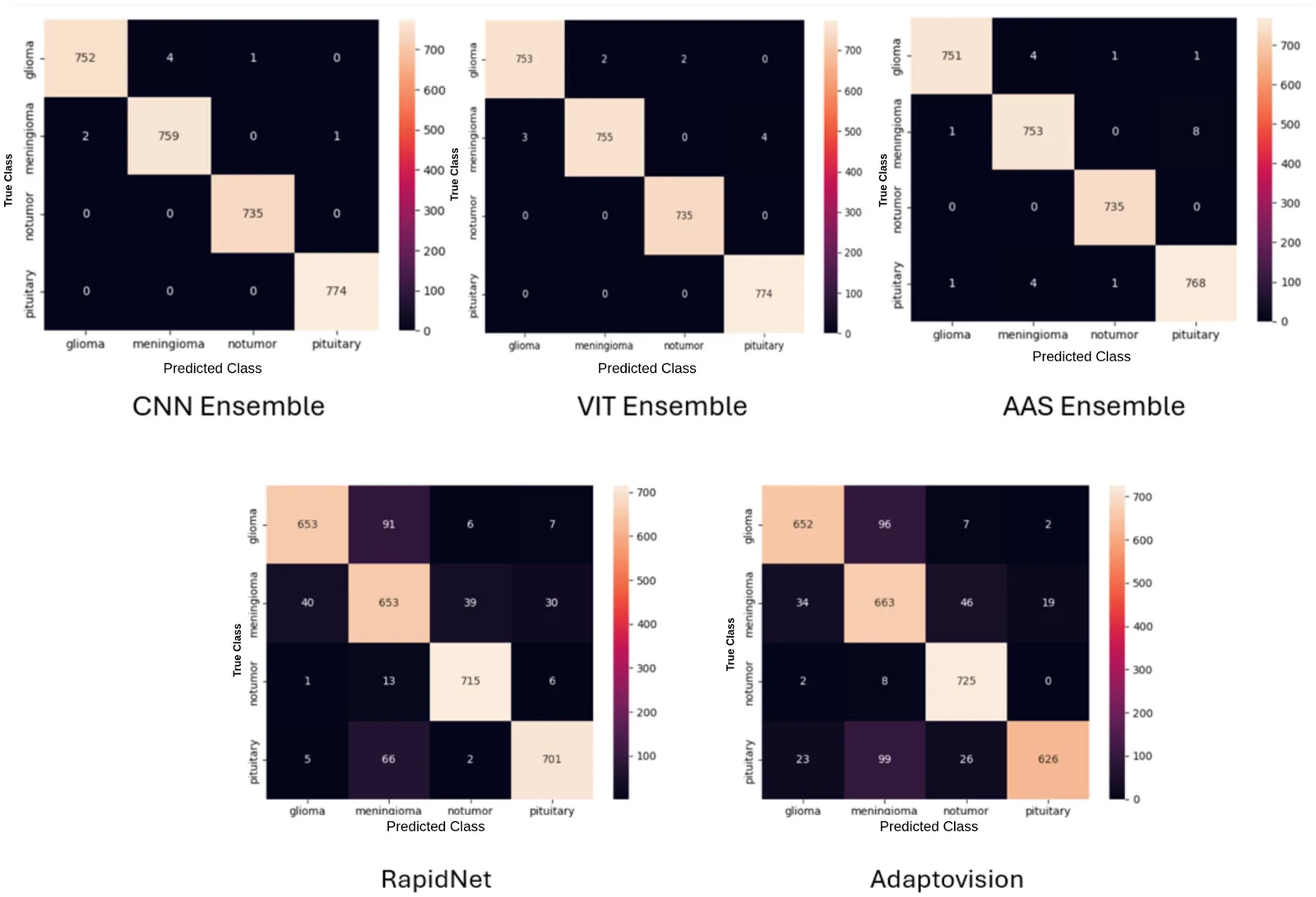
Confusion matrices on the internal merged test set for the evaluated models.

Figure 3 shows the confusion matrices on the internal merged test set. The CNN and ViT ensemble baselines classify nearly all samples correctly, with only minor confusion between closely related classes. NeuroTrustNet achieves similarly strong internal performance, indicating that adaptive fusion preserves discriminative power while integrating heterogeneous representations.

**Figure 4:**
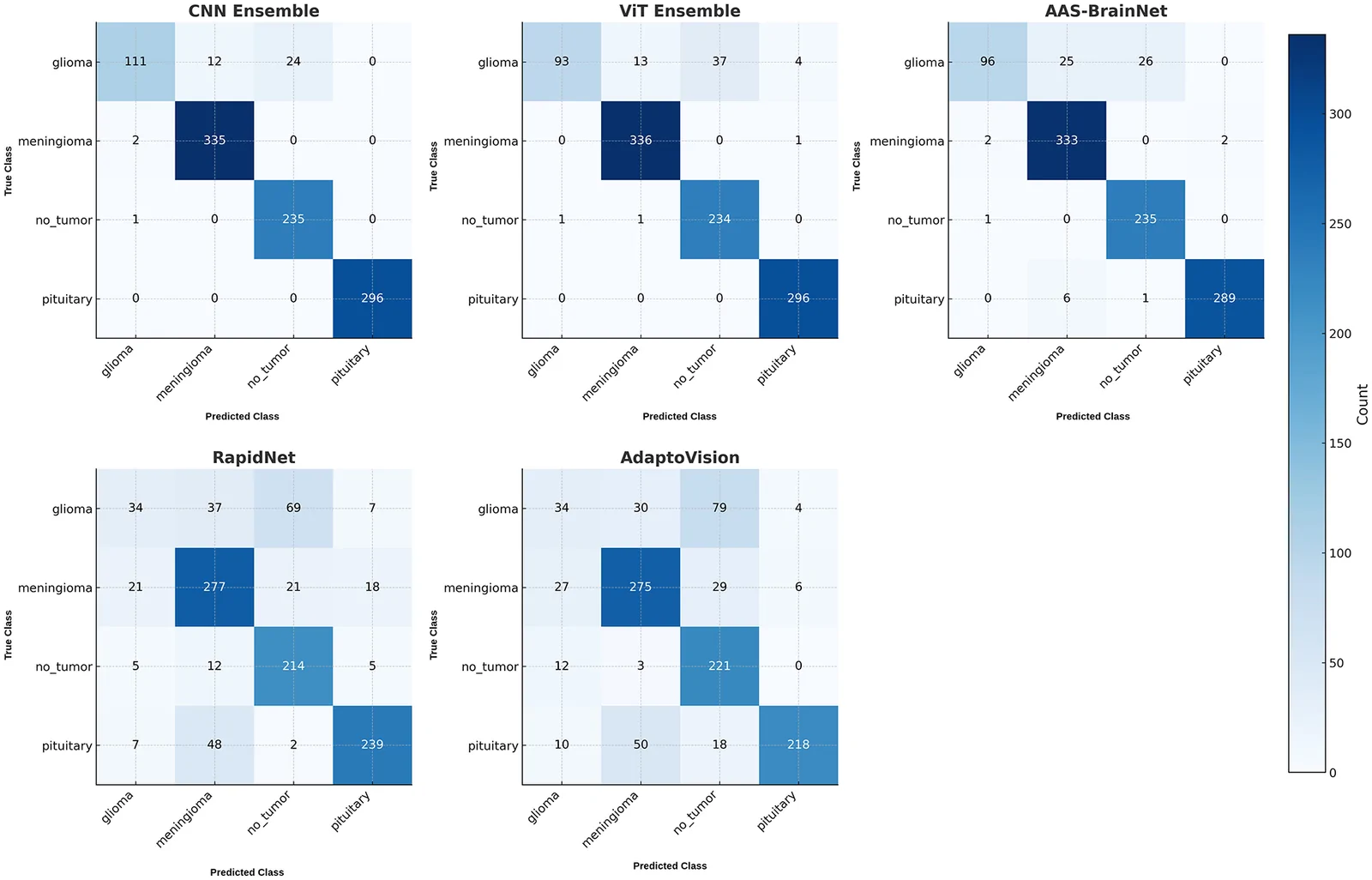
Confusion matrices on the external Sherif dataset for the evaluated models.

On the external Sherif dataset (Figure 4), all models experience a noticeable drop in performance, confirming the presence of cross-dataset domain shift. Nevertheless, NeuroTrustNet maintains relatively balanced confusion patterns across classes and reduces severe off-diagonal errors compared with the lightweight baselines. Glioma remains the most challenging class for all models, which is consistent with its broader visual heterogeneity.

**Figure 5:**
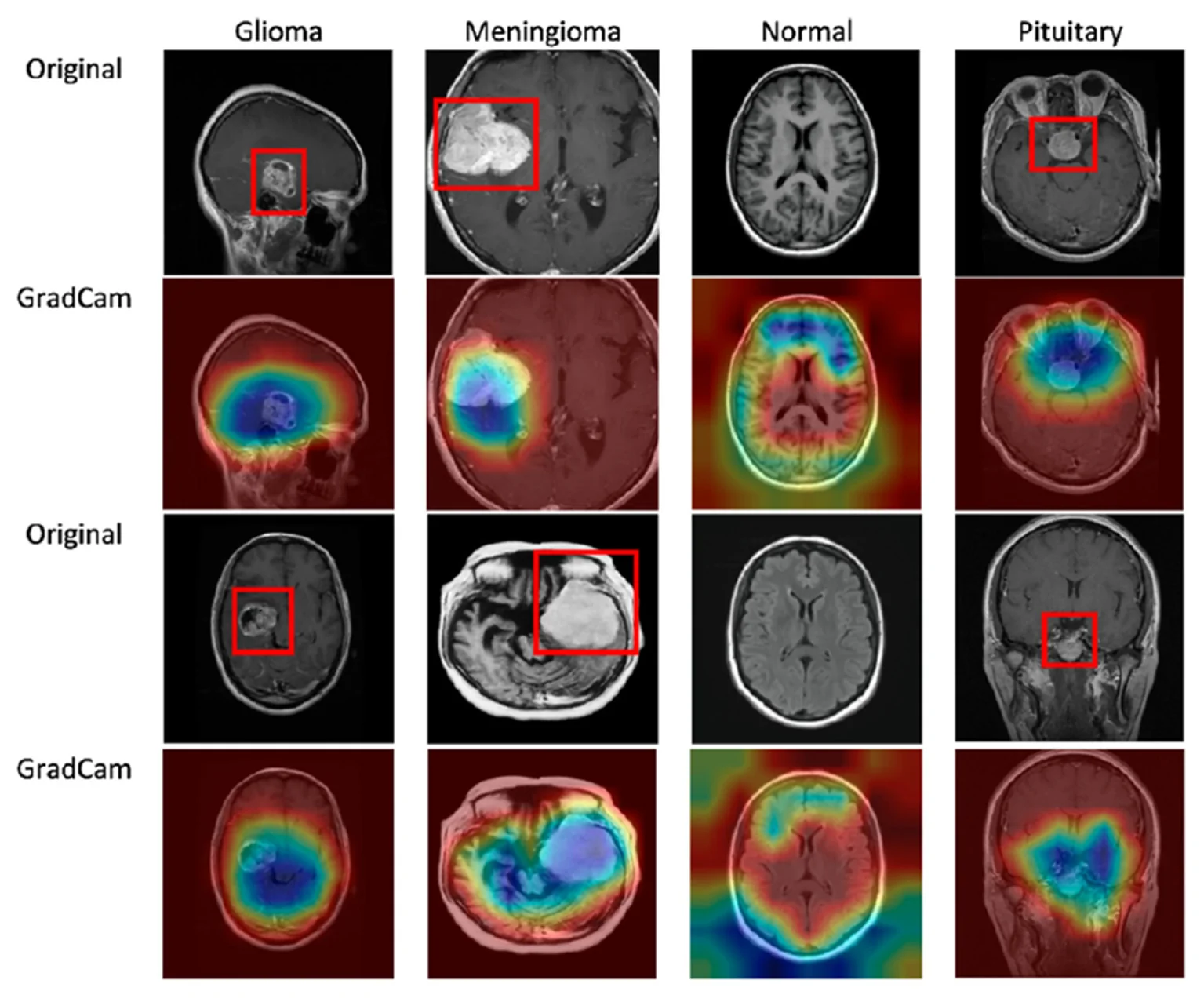
Representative Grad-CAM visualizations for glioma, meningioma, no-tumor, and pituitary samples.

Figure 5 presents representative Grad-CAM visualizations. Across model families, the highlighted regions generally align with anatomically meaningful tumor-related structures rather than irrelevant background patterns. NeuroTrustNet preserves coherent attention maps while integrating CNN, ViT, and radiomic information, suggesting that its predictions remain grounded in clinically plausible image regions.

Taken together, the confusion-matrix and visual explanation analyses indicate that NeuroTrustNet achieves a favorable balance between predictive robustness and interpretability, particularly when compared with lightweight alternatives.

Although the proposed evidence-grounded prompting strategy reduces the likelihood of unsupported statements, the LLM may still generate imperfect explanations. Future work will include clinician-based evaluation and quantitative assessment of explanation faithfulness.

### 4.6 Error analysis

To better understand the limitations of the proposed framework, representative misclassified cases were examined from the external Sherif dataset. Most classification errors occurred between glioma and meningioma samples, particularly for tumors exhibiting ambiguous boundaries, low image contrast, or overlapping structural characteristics. A small number of pituitary tumors were also misclassified when the lesion occupied only a limited image region, reducing the discriminative information available to both CNN and ViT branches. These observations suggest that challenging anatomical appearances and domain shifts between datasets remain important factors affecting model performance. Future work will investigate uncertainty-aware learning and multi-center clinical datasets to improve robustness under such challenging conditions.

### 4.7 Web-based clinical interface

**Figure 6:**
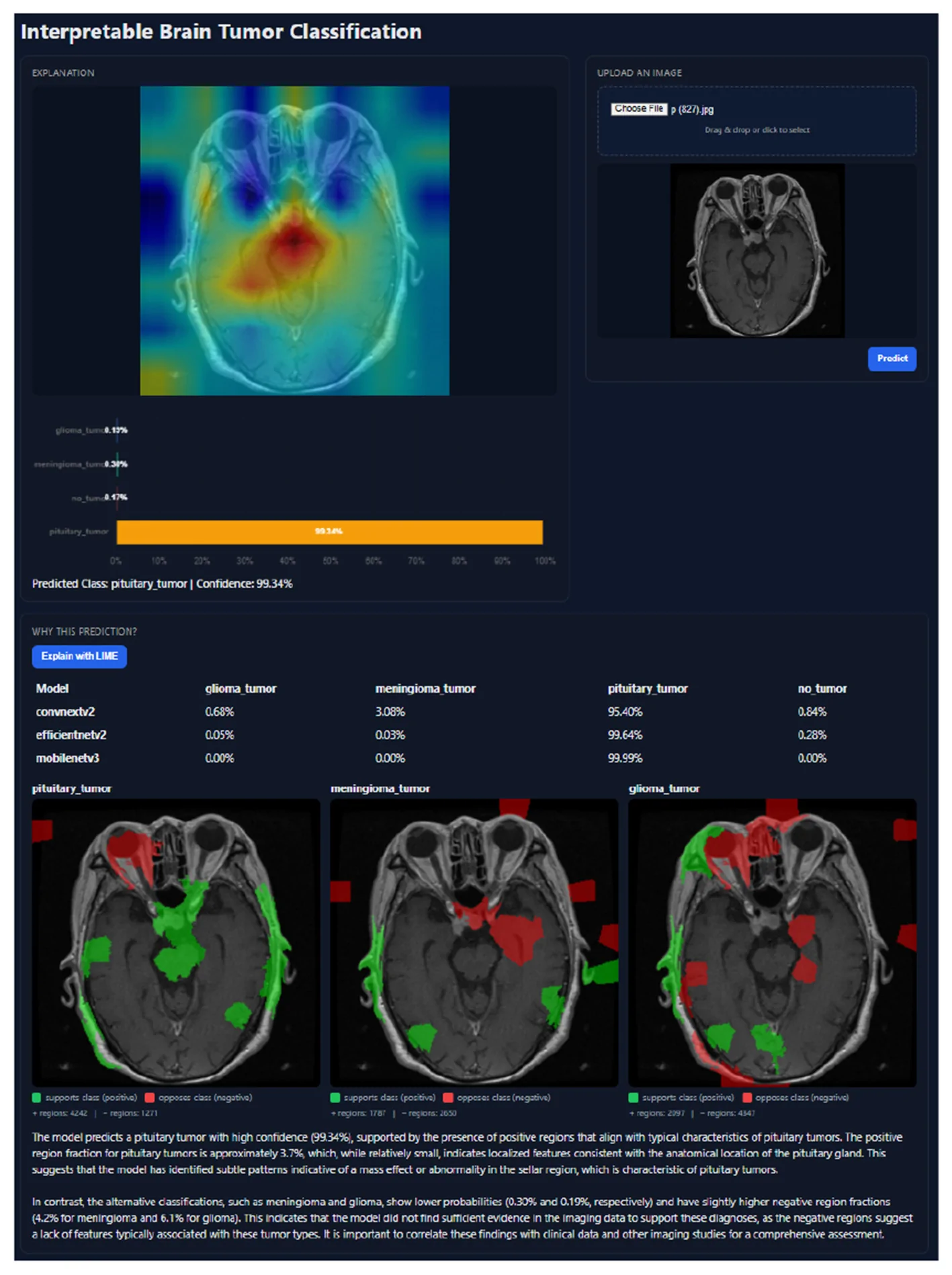
Web-based interface for NeuroTrustNet, integrating classification output, confidence estimation, visual explanation, and textual interpretation.

To demonstrate practical usability, NeuroTrustNet was integrated into a web-based decision-support interface, shown in Figure 6. The interface accepts MRI slices as input and returns four outputs: predicted class, confidence score, visual explanation maps, and an LLM-generated textual summary grounded in visual evidence.

The system is implemented using a lightweight client–server design. The front-end supports image upload and result visualization, while the backend performs preprocessing, branch inference, AAS fusion, and explanation generation. Average end-to-end response times remained suitable for interactive use on an RTX A4000 GPU, indicating that the framework can support near-real-time analysis despite its multimodal design.

Although NeuroTrustNet is not intended to replace clinical decision-making, it can serve as a computer-aided diagnostic support tool within the radiological workflow. In a typical deployment scenario, an MRI scan is first analyzed by NeuroTrustNet, which provides the predicted tumor class together with Grad-CAM visualizations and LLM-generated textual explanations. The radiologist then reviews both the original MRI and the AI-generated evidence before making the final clinical decision. This human-in-the-loop workflow enables clinicians to benefit from improved diagnostic consistency while maintaining full responsibility for the final interpretation and treatment planning.

### 4.8 Comparison with prior studies

Table 9 compares the evaluated models with representative prior studies reported on related brain tumor MRI datasets. Many previous studies report strong performance on different benchmark datasets using distinct preprocessing pipelines, train–test split strategies, and evaluation protocols. Therefore, Table 9 is intended as a contextual comparison rather than a direct benchmark comparison. The reported performance values should be interpreted cautiously, as differences in experimental settings may substantially influence the reported metrics.

**Table 9:** Contextual comparison with representative prior studies on brain tumor MRI classification.

Model	Dataset(s)	Accuracy (%)	F1-score (%)
Optimized ResNet101 (Rasheed et al., 2025)	Sartaj	98.73	–
InceptionV3 (Almadhoun and Abu-Naser, 2022)	Pradeep	99.88	–
GAN+CNN (Melekoodappattu et al., 2023)	Sherif	99.63	–
TumorAwareNet (Bodapati and Balaji, 2024)	Sherif	93.50	91.25
EfficientNet-B7 (Ghosh et al., 2024)	Sartaj, Sherif	97.0, 96.0	96.0, 97.0
ConvBiFuseNet (Liu et al., 2025)	Sartaj, Sherif	98.73, 97.91	98.77, 97.74
Xception (Abbasi, 2024)	Sartaj	97.60	97.90
EfficientNet-B1 (Ishaq et al., 2025)	Masoud, Sartaj	97.0, 92.0	97.0, 91.0
MobileNet (Islam et al., 2023)	Masoud	96.00	95.75
CNN-ML Ensemble (Celik and Inik, 2024)	Masoud	96.00	95.91
RanMerFormer (Wang et al., 2024)	Masoud	99.17	–
Hybrid ViT-DNN (Dixon et al., 2024)	Masoud	99.19	–
PDSCNN-RRELM (Nahiduzzaman et al., 2025)	Masoud	99.22	99.30
NeuroNet19 (Haque et al., 2024)	Masoud	99.30	–
CNN ensemble baseline	Masoud, Sartaj, Pradeep	99.73	99.73
ViT ensemble baseline	Masoud, Sartaj, Pradeep	99.63	99.63
**NeuroTrustNet (AAS)**	**Masoud, Sartaj, Pradeep**	**99.30**	**99.31**

Bold values highlight the proposed NeuroTrustNet/AAS configuration.

Within this context, the CNN and ViT ensemble baselines achieve competitive predictive performance, while NeuroTrustNet offers a more deployment-oriented balance between accuracy, interpretability, and computational efficiency. Consequently, the comparison is intended to highlight the relative positioning of the proposed framework within the existing literature rather than to claim direct numerical superiority over studies conducted under different experimental conditions. This is particularly relevant for real-world settings, where inference cost and transparency are important alongside classification accuracy.

### 4.9 Limitations

Despite the encouraging results, several limitations should be acknowledged. First, this study was conducted using 2D MRI slices rather than full volumetric MRI scans, which may limit the ability to capture three-dimensional tumor characteristics. Second, although NeuroTrustNet was evaluated on an independent external dataset, all experiments were performed using publicly available benchmark datasets. Consequently, the reported performance may not fully reflect the diversity of real-world clinical MRI acquisitions obtained from different hospitals, scanners, imaging protocols, and patient populations. Future work will therefore focus on large-scale multi-center institutional datasets and prospective clinical studies to further assess the robustness and generalizability of the proposed framework in routine clinical environments. Third, the handcrafted radiomic branch relies on PCA and GWO for dimensionality reduction and feature selection, which may not fully capture complex nonlinear feature interactions. Fourth, the proposed framework has not yet been validated within prospective clinical workflows and should therefore be regarded as a decision-support research framework rather than a standalone diagnostic system. Finally, although the explainability module combines Grad-CAM visualizations, LIME explanations, and LLM-generated textual summaries to improve model transparency, its effectiveness was evaluated qualitatively without quantitative localization metrics or formal assessment by expert radiologists. Consequently, the interpretability results should be considered illustrative rather than clinically validated. Future work will include clinician-based user studies, quantitative localization metrics, and workflow-oriented evaluations to further establish the clinical usefulness and reliability of the proposed explainability framework. Furthermore, although evidence-grounded prompting constrains the large language model (LLM) using Grad-CAM visualizations, prediction confidence, and class probabilities, hallucinated or clinically unsupported textual descriptions may still occur. Consequently, the generated explanations should be regarded as supplementary decision-support information rather than definitive clinical interpretations. Formal evaluation of explanation faithfulness, clinical usefulness, and agreement among radiologists therefore represents an important direction for future work.

## 5 Conclusion

This study presented *NeuroTrustNet*, a multimodal brain tumor classification framework designed to balance predictive performance, computational efficiency, and interpretability under heterogeneous MRI conditions. The proposed system integrates CNN-based local feature extraction, ViT-based global contextual modeling, and handcrafted radiomic descriptors through the proposed Adaptive Attention Stacking (AAS) mechanism. Experimental results on a large multi-dataset corpus demonstrated that although high-capacity ensemble baselines achieved the highest classification accuracy, NeuroTrustNet maintained competitive predictive performance on both internal and external evaluation datasets while substantially reducing computational cost, thereby providing a practical balance between predictive performance, computational efficiency, and explainability for deployment-oriented medical AI systems. Furthermore, the integration of evidence-grounded explainability through Grad-CAM visualizations and LLM-generated textual summaries enhances the transparency and interpretability of model predictions without influencing the underlying decision-making process. Overall, the results demonstrate the potential of multimodal adaptive fusion as an effective strategy for robust and interpretable brain tumor classification under cross-dataset variability. Rather than solely maximizing benchmark accuracy, NeuroTrustNet aims to achieve a balanced trade-off among predictive performance, computational efficiency, and interpretability. Future work will focus on volumetric MRI analysis, integration of clinical metadata, stronger domain generalization strategies, and comprehensive validation using large-scale multi-center clinical datasets collected from diverse hospitals, MRI scanners, and imaging protocols to further assess the robustness, generalizability, and clinical applicability of the proposed framework under real-world diagnostic conditions.

## Data Availability

Publicly available datasets were analyzed in this study. This data can be found here: kaggle.com.
